# Improved prediction and characterization of anticancer activities of peptides using a novel flexible scoring card method

**DOI:** 10.1038/s41598-021-82513-9

**Published:** 2021-02-04

**Authors:** Phasit Charoenkwan, Wararat Chiangjong, Vannajan Sanghiran Lee, Chanin Nantasenamat, Md. Mehedi Hasan, Watshara Shoombuatong

**Affiliations:** 1 Modern Management and Information Technology, College of Arts, Media and Technology, Chiang Mai University, Chiang Mai, 50200 Thailand; 2 Pediatric Translational Research Unit, Department of Pediatrics, Faculty of Medicine, Ramathibodi Hospital, Mahidol University, Bangkok, 10400 Thailand; 3 Department of Chemistry, Centre of Theoretical and Computational Physics, Faculty of Science, University of Malaya, 50603 Kuala Lumpur, Malaysia; 4 Center of Data Mining and Biomedical Informatics, Faculty of Medical Technology, Mahidol University, Bangkok, 10700 Thailand; 5 Department of Bioscience and Bioinformatics, Kyushu Institute of Technology, 680-4 Kawazu, Iizuka, Fukuoka 820-8502 Japan

**Keywords:** Computational biology and bioinformatics, Computational models, Machine learning, Protein function predictions

## Abstract

As anticancer peptides (ACPs) have attracted great interest for cancer treatment, several approaches based on machine learning have been proposed for ACP identification. Although existing methods have afforded high prediction accuracies, however such models are using a large number of descriptors together with complex ensemble approaches that consequently leads to low interpretability and thus poses a challenge for biologists and biochemists. Therefore, it is desirable to develop a simple, interpretable and efficient predictor for accurate ACP identification as well as providing the means for the rational design of new anticancer peptides with promising potential for clinical application. Herein, we propose a novel flexible scoring card method (FSCM) making use of propensity scores of local and global sequential information for the development of a sequence-based ACP predictor (named iACP-FSCM) for improving the prediction accuracy and model interpretability. To the best of our knowledge, iACP-FSCM represents the first sequence-based ACP predictor for rationalizing an in-depth understanding into the molecular basis for the enhancement of anticancer activities of peptides via the use of FSCM-derived propensity scores. The independent testing results showed that the iACP-FSCM provided accuracies of 0.825 and 0.910 as evaluated on the main and alternative datasets, respectively. Results from comparative benchmarking demonstrated that iACP-FSCM could outperform seven other existing ACP predictors with marked improvements of 7% and 17% for accuracy and MCC, respectively, on the main dataset. Furthermore, the iACP-FSCM (0.910) achieved very comparable results to that of the state-of-the-art ensemble model AntiCP2.0 (0.920) as evaluated on the alternative dataset. Comparative results demonstrated that iACP-FSCM was the most suitable choice for ACP identification and characterization considering its simplicity, interpretability and generalizability. It is highly anticipated that the iACP-FSCM may be a robust tool for the rapid screening and identification of promising ACPs for clinical use.

## Introduction

Anticancer peptides (ACPs) are small peptides exerting selective and toxic properties toward cancer cells. Owing to its inherent high penetration, high selectivity and ease of modification, synthetic peptide-based drugs and vaccines^1–3^ represents a promising class of therapeutic agents. Designed ACPs can improve affinity, selectivity and stability for enhancing cancer cell elimination. The influence of amino acid residues towards the anticancer activity of ACPs is dependent on cationic, hydrophobic and amphiphilic properties with helical structure to drive cell permeability. Particularly, cationic amino acid residues (i.e., lysine, arginine, and histidine) can disrupt and penetrate the cancer cell membrane to induce cytotoxicity whereas anionic amino acids (i.e., glutamic and aspartic acids) affords antiproliferative activity against cancer cells. Furthermore, hydrophobic amino acid residues (i.e., phenylalanine, tryptophan, and tyrosine) exerts their effect on the cancer cytotoxic activity^1,4,5^. Moreover, the secondary structure of ACPs that is formed by cationic and hydrophobic amino acids, plays a crucial role in peptide-cancer cell membrane interaction that inherently leads to cancer cell disruption and death^1,6^. Therefore, it is desirable to develop a simple, interpretable and efficient predictor for achieving accurate ACP identification as well as facilitating the rational design of new anticancer peptides with promising clinical applications.

In the past few years, most methods in existence were developed via the use of machine learning (ML) and statistical methods as applied on peptide sequence information for discriminating ACPs from non-ACPs^7–23^. More details of those existing methods are summarized in two comprehensive review papers^2,3^. Amongst the various types of ML approaches, both support vector machine (SVM) (i.e. AntiCP^8^, Hajisharifi et al.’s method^9^, ACPP^24^, iACP^10^, Li and Wang’s method^11^, iACP-GAEnsC^12^, TargetACP^14^ and ACPred^19^) and the ensemble approach (i.e. MLACP^13^, ACPred^19^, PTPD^21^, ACP-DL^22^, PEPred-Suite^20^, ACPred-FL^15^, ACPred-Fuse^18^, PPTPP^23^ and AntiCP_2.0^25^) were widely used to develop ACP predictors. As summarized in a recent review^2^, we could see that TargetACP has been developed by integrating the split amino acid composition and pseudo position-specific scoring matrix descriptors^14^, which was shown to outperform SVM-based predictors^8–12,19,24^. In the meanwhile, the state-of-the-art ensemble methods comprising PEPred-Suite^20^ and ACPred-Fuse^18^ provided the highest prediction accuracies as evaluated on the dataset collected by Rao et al.^18^. In ACPred-Fuse, it was developed using random forest (RF) model in conjunction with 114 feature descriptors. And then, a total of 114 RF models were trained to generate class information and probabilistic information used for developing a final model. Most recently, Agrawal et al. proposed an updated version of AntiCP called AntiCP2.0 and also provided two high-quality benchmark datasets (i.e. main and alternative datasets) having the largest number of peptides. AntiCP2.0 was developed by extremely randomized trees (ETree) algorithm with amino acid composition (AAC) and dipeptide composition (DPC). On the basis of independent test results reported by the prior work of AntiCP2.0, it can be noticed that AntiCP2.0 was superior to other existing ACP predictors (e.g. AntiCP^8^, iACP^10^, ACPred^19^, ACPred-FL^15^, ACPred-Fuse^18^, PEPred-Suite^20^). All in all, much progress has been achieved in existing methods. Nevertheless, two potential drawbacks of existing ACP predictors motivated us to develop a new ACP predictor in this study. First, their interpretable mechanisms are not easily understood and implemented by the viewpoint of biologists and biochemists. Existing ACP models do not provide a straight-forward explanation on the underlying mechanism of the biological activity of what constitute ACPs. Meanwhile, a simple and easily interpretable models is more useful in a further analysis of characteristics of anticancer activities of peptides. Second, their accuracy and generalizability still require improvement.

**Figure 1:**
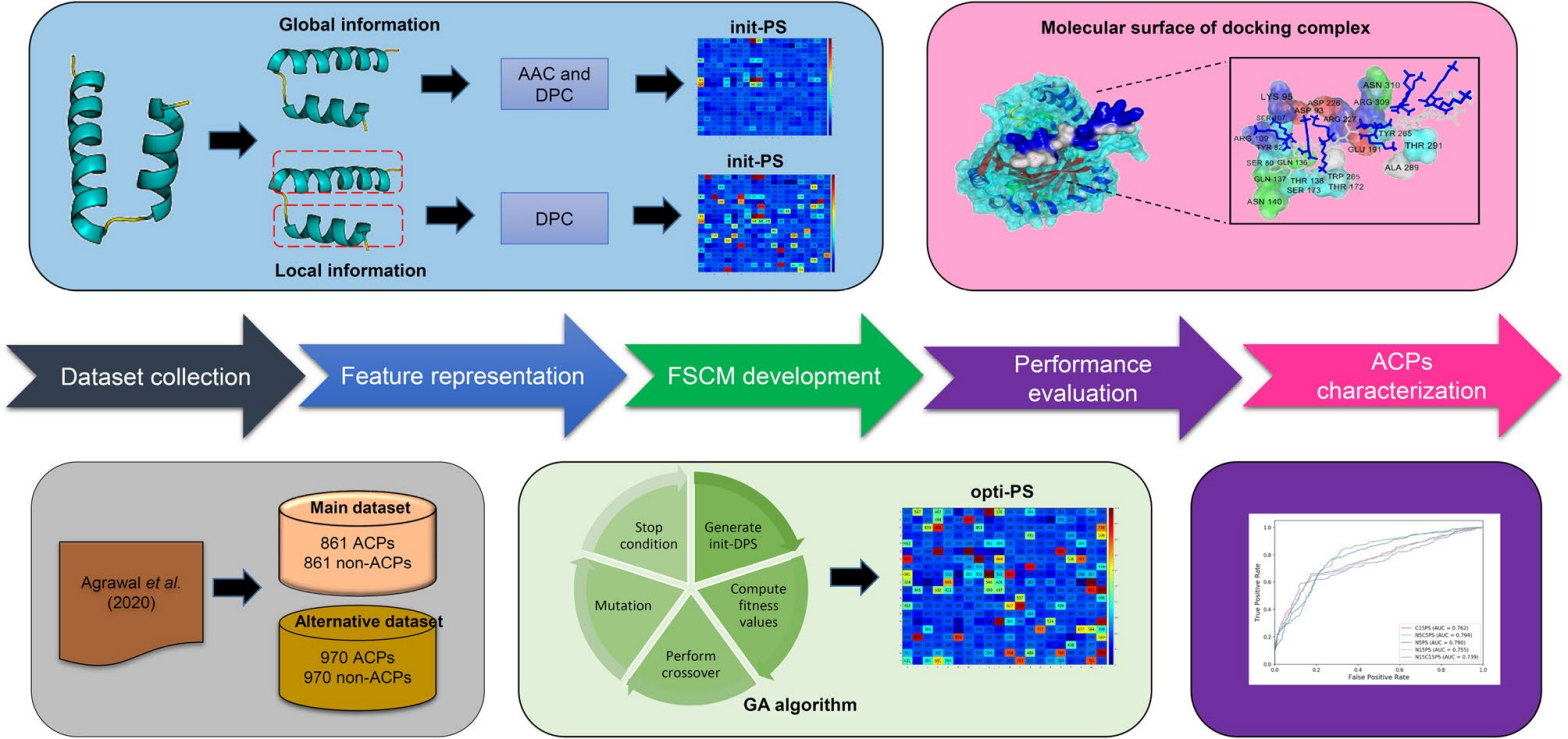
System flowchart of the proposed iACP-FSCM. There are five main steps are involved in the development of proposed iACP-FSCM as follows: (i) preparing the training and independent datasets, (ii) calculating the initial propensity score (init-PS) using a statistical approach, (iii) estimating the optimized propensity score (opti-PS) using a genetic algorithm (GA), (iv) evaluating the prediction ability of iACP-FSCM, (v) ACPs characterization using the propensity scores and docking approach.

In consideration of these problems, we propose herein the development of a novel ML-based predictor called the iACP-FSCM for further improving the prediction accuracy as well as shedding light on characteristics governing anticancer activities of peptides. The conceptual framework of the iACP-FSCM approach proposed herein for predicting and analyzing ACPs is summarized in Fig. 1. The major contributions of iACP-FSCM for predicting and characterizing ACPs can be summarized as follows. Firstly, we proposed herein a novel, flexible scoring card method (FSCM) for effective and simple prediction and characterization of peptides affording anticancer activity using only sequence information. The FSCM method is an updated version of the SCM method developed by Huang et al.^26^ and Charoenkwan et al.^27^ by making use of propensity scores of both local and global sequential information. Secondly, unlike the rather complex classification mechanisms as afforded by state-of-the-art ensemble approaches^15,18,20^, the iACP-FSCM method proposed herein identifies ACPs using only weighted-sum scores between the composition and propensity scores, which is easily understood and implemented by biologists and biochemists. Thirdly, the FSCM-derived propensity scores can be adopted to identify informative physicochemical properties (PCPs) that may provide crucial information pertaining to local and global properties of ACPs. Finally, comparative results revealed that iACP-FSCM outperformed those of state-of-the-art ACP predictors for ACP identification and characterization. The iACP-FSCM webserver presented herein has been demonstrated to be robust as deduced from its superior prediction accuracy, interpretability and publicly availability, which is instrumental in helping biologists in their identification of ACPs with potential bioactivities. Furthermore, the proposed FSCM method has great potential for estimating the propensity scores of amino acids and dipeptides that can be used to predict and analyze various bioactivities of peptides such as haemolytic peptides^28^, antihypertensive peptides^29^ and antiviral peptides^20,23^.

## Materials and methods

### Benchmark datasets

In order to make a fair comparison with existing methods, the most recent and high-quality benchmark datasets (i.e. main and alternative datasets) collected from the work of AntiCP_2.0^25^ were used in the development and validation of the iACP-FSCM model proposed herein. Both datasets can be downloaded from https://webs.iiitd.edu.in/raghava/anticp2/download.php. The main dataset consists of 861 experimentally validated ACPs and 861 AMPs while the alternative dataset consists of 970 experimentally validated 970 ACPs and 970 random peptides from protein in SwissProt. All peptides on main and alternative datasets were unique. To avoid overestimation in the prediction model, the main and alternative dataset were randomly divided as the training (named MAIN-TR and ALTER-TR) and independent sets (named MAIN-TS and ALTER-TS) using the 80:20 ratio. Further details regarding the construction of the main and alternative datasets is provided in the original work of AntiCP_2.0^25^.

### Protein feature representation

In this study, we employed 11 feature classes generated from 3 different feature encodings using AAC, DPC and terminus compositions for representing peptide sequences as feature vectors with fixed length. Herein, we briefly describe each feature encoding definition in forthcoming subsections.

#### Amino acid composition

AAC is the proportion of any amino acid in a given peptide **P**. AAC descriptor can be represented as formulated by:

1 $$ {\text{AAC}}\left( {\mathbf{P}} \right) = ({\text{aac}}_{1} ,{\text{aac}}_{2} , \ldots , {\text{aac}}_{20} ) $$

where aac_i_ is the normalized composition of the *i*th amino acid (aa_i_). The dimension of AAC descriptor is 20.

#### Dipeptide composition

DPC is the proportion of any two adjacent amino acids (aa_i_, aa_j_) in a given peptide **P**. DPC descriptor can be represented as formulated by:

2 $$ {\text{DPC}}\left( {\mathbf{P}} \right) = ({\text{dpc}}_{1} ,{\text{dpc}}_{2} , \ldots , {\text{dpc}}_{400} ) $$

where dpc_i_ is the normalized composition of the *i*th dipeptide (dp_i_). The dimension of DPC descriptor is 400.

#### Composition on terminal region

Keeping in mind that the information on N- and C-terminus are important in the biological activity of peptides^7,8,19,30–33^, we thus calculated the DPC information using the first 5, 10 and 15 residues from the N (i.e. N5, N10 and N15, respectively) and C terminus (i.e. C5, C10 and C15, respectively). In addition, we also joined these terminus sequence and their DPC as follows: N5C5, N10C10 and N15C15. The dimension of DPC on terminal region descriptor is 400.

### Flexible scoring card method

The original SCM method uses only the global sequential information (i.e. 20 amino acids (APS) and 400 dipeptides (DPS) propensity scores) for prediction and analysis of proteins^26,27^. Inspired by this method, we developed and implemented a novel flexible SCM-based method called FSCM to further improve the prediction accuracy and interpretability by utilizing both local and global sequential information of peptides. DPS was used to provide local sequence information as they were found to yield better prediction performance and provide more information than APS. Particularly, the FSCM method estimated the propensity scores of 400 dipeptides on N- (N5PS, N10PS and N15PS) and C- (C5PS, C10PS and C15PS) terminus as well as their joint terminus sequences (N5C5PS, N10C10PS and N15C15PS). In the proposed iACP-FSCM, we built 11 FSCM models obtained using different 11 propensity scores of amino acids, dipeptides and dipeptide on N- and C-terminus for main and alternative dataset each. Below, we briefly describe the basic concepts and the optimization procedures of C15PS on main dataset, since the other types of propensity scores can be estimated in the same procedure without significant modifications.

**Phase 1:** Preparing the training (MAIN-TR) and independent (MAIN-TS) datasets for the development and evaluation of the proposed model as described above.

**Phase 2:** Calculating the initial propensity score of 400 dipeptides on the first 15 residues from the C terminus (init-C15PS). According to Charoenkwan et al.^34–37^, the init-C15PS is estimated, as follows:

*Step 1:* Computing the frequency of all 400 dipeptides found in ACP and non-ACP. For example, the frequency of KK presented in ACP and non-ACP classes consisted of 280 and 40, respectively.

*Step 2:* Calculating the ratio between each dipeptide by the total number of dipeptides for ACP and non-ACP classes. For example, the total number of dipeptides in ACP and non-ACP classes were 450 and 200, respectively. Therefore, normalized compositions of KK dipeptide in ACP and non-ACP classes (called NPS^+^ and NPS^-^, respectively) were 0.622 and 0.2, respectively.

*Step 3:* Computing the score of each dipeptide by subtracting NPS^+^ from NPS^-^. For example, the score of DE dipeptide is 0.422 (0.622–0.2).

*Step 4:* Normalizing the score of each dipeptide into the range of 0–1000.

**Phase 3:** Estimating the optimized propensity score of 400 dipeptides (opti-C15PS) and the threshold value using the GA algorithm^37^. More details of the GA algorithm used in this study can be found in the Supplementary information S1. To obtain the best opti-C15PS, the corresponding threshold value are subjected to the fitness function^26,27^ whereby the prediction performance in terms of the AUC ($W_{1}$) and the Pearson’s correlation coefficient ($W_{2}$) between init-C15PS and opti-C15PS are linearly combined and assessed by a tenfold cross-validation procedure:

3 $$ {\text{F}}\left( {{\text{opti}} - {\text{C}}15{\text{PS}}} \right) = W_{1} \times {\text{AUC}} + { }W_{2} \times {\text{R}} $$

where values of $W_{1}$ and $W_{2}$ are 0.9 and 0.1, respectively. Furthermore, weights for $W_{1}$ and $W_{2}$ were set based on our previous studies^27,34–37^.

**Phase 4:** Computing the propensity scores of 20 amino acids using the opti-C15PS from Phase 3. Taking Lys as an example, the propensity score for Lys is calculated by averaging the propensity scores of 40 dipeptides containing Lys.

**Phase 5:** Predicting an unknown peptide (*P*) by using the scoring function S(*P*) and the opti-C15PS from Phase 3. A query peptide *P* is predicted to be ACP if S(*P*) is greater than the threshold value, otherwise *P* is predicted to be a non-ACP.

4 $$ S\left( P \right) = \mathop \sum \limits_{i = 1}^{400} DP_{i} PS_{i} $$

where $DP_{i}$ and $PS_{i}$ represent the occurrence frequency and propensity score of the *i*^th^ dipeptide from the opti-C15PS, respectively, where *i* = 1, 2, 3, …, 400.

**Phase 6:** Evaluating the prediction ability of the model by using four widely used metrics for binary classification problems consisting of accuracy (Ac), sensitivity (Sn), specificity (Sp) and Matthew’s coefficient correlation (MCC)^38,39^. Receiver operating characteristic (ROC) curves were plotted to further investigate the prediction performance of the proposed model using threshold-independent parameters. Further details on the definition of these metrics can be found in the Supplementary data S1.

### Characterization of anticancer activities of peptides

The propensity score of 20 amino acids are informative PCPs that were employed for providing an in-depth understanding on the basis and important factors governing the anticancer activity. Particularly, propensity scores of each amino acid reflect its influence on the biological, functional and structural properties of peptides. It is well-known that PCPs are one of the most intuitive feature descriptors associated with biophysical and biochemical reactions. Informative PCPs were determined from the iACP-FSCM method according to three main steps. Firstly, PCPs having not applicable (NA) as their amino acid indices were excluded and this resulted in a total of 531 PCPs^40^ that were further used in this study. Secondly, the Pearson’s correlation coefficient (R) value between the propensity scores of amino acids with those of 531 PCPs were calculated. Finally, PCPs with an absolute R value greater than 0.5 will be selected as candidate PCPs for further analysis.

### Reproducible research

To ensure the repeatability and reproducibility of proposed models, all codes and the benchmark datasets (i.e. main and alternative datasets) are available on GitHub at https://github.com/Shoombuatong/Dataset-Code/tree/master/iACP-FSCM.

## Results and discussion

### Performance evaluation on main dataset

**Figure 2:**
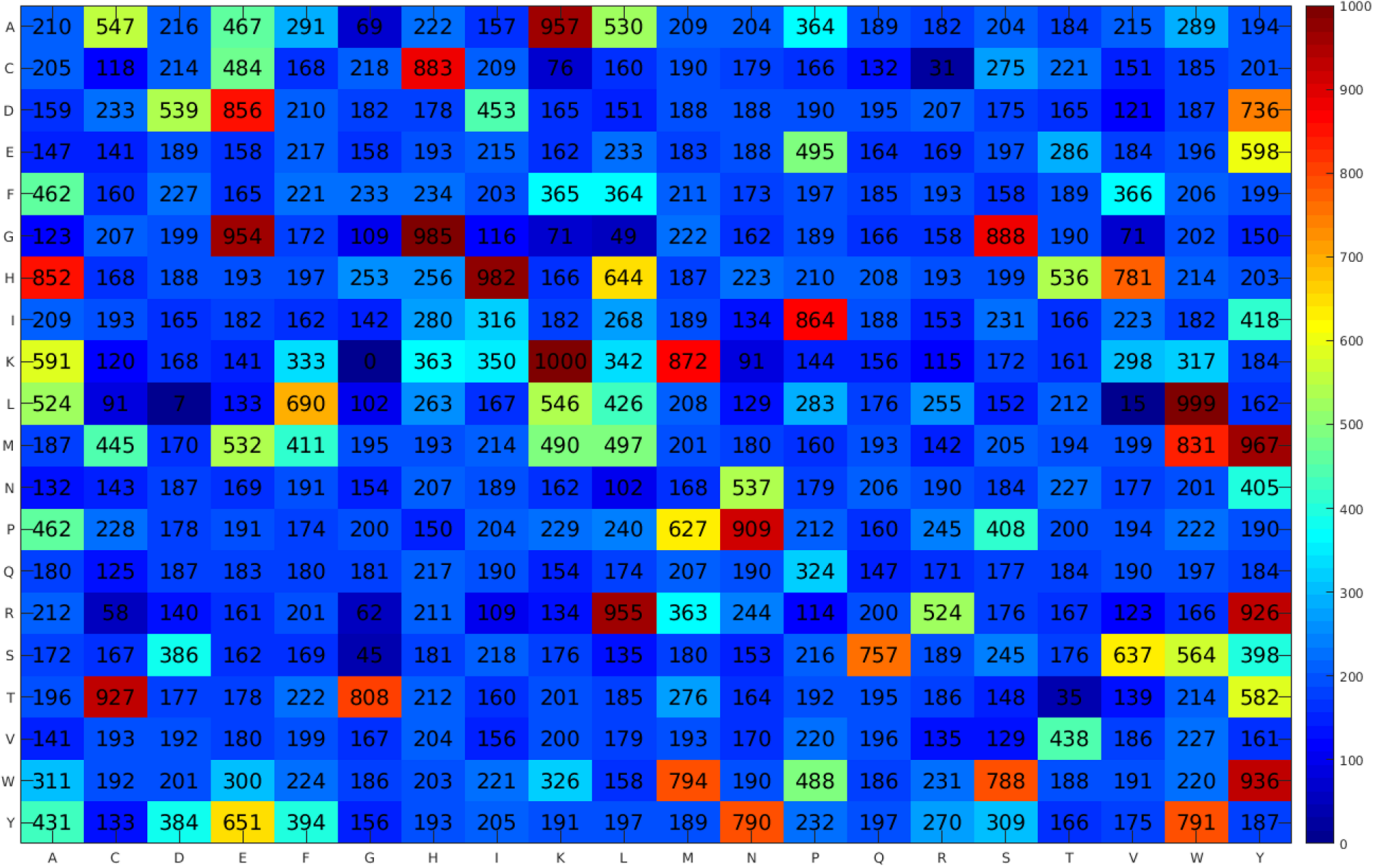
Heatmap of amino acids propensity scores obtained from the proposed iACP-FSCM.

In this study, we employed 11 feature classes generated from 3 different feature encodings using AAC, DPC and terminus compositions (i.e. N5, C5, N5C5, N10, C10, N10C10, N15, C15 and N15C15). Particularly, this led to the generation of 11 types of propensity scores (i.e. APS, DPS, N5PS, C5PS, N5C5PS, N10PS, C10PS, N10C10PS, N15PS, C15PS and N15C15PS). To examine which types of propensity scores are beneficial for distinguishing ACPs from non-ACPs, we performed performance comparisons of different types of propensity scores via tenfold cross-validation and independent tests on main dataset. For each type of propensity scores, 10 sets of propensity scores were generated by the GA algorithm and then used in the development of 10 different FSCM classifiers. Tables 1 and 2 lists the best prediction results as derived from optimal sets for each type of propensity scores via tenfold cross-validation and independent tests, respectively (Fig. 2).

**Table 1:** Cross-validation results of FSCM models with various types of sequence features as evaluated on the main dataset.

Method	Threshold	Fitness score	Ac	Sn	Sp	MCC	AUC
APS	399	0.742	0.668	0.624	0.711	0.338	0.686
DPS	285	0.420	0.726	0.675	0.778	0.456	0.754
N5PS	218	0.660	0.752	0.794	0.710	0.507	0.791
C5PS	266	0.566	0.710	0.715	0.705	0.421	0.729
N5C5PS	224	0.568	0.750	0.831	0.671	0.508	0.794
N10PS	219	0.429	0.736	0.733	0.739	0.473	0.771
C10PS	248	0.444	0.739	0.739	0.739	0.480	0.766
N10C10PS	225	0.482	0.732	0.728	0.736	0.465	0.764
N15PS	394	0.478	0.746	0.638	0.829	0.480	0.755
C15PS	311	0.401	0.754	0.656	0.829	0.496	0.762
N15C15PS	394	0.424	0.743	0.570	0.877	0.478	0.739

**Table 2:** Independent test results of FSCM models with various types of sequence features as evaluated on the main dataset.

Method	Threshold	Fitness score	Ac	Sn	Sp	MCC	AUC
APS	399	0.742	0.701	0.669	0.733	0.402	0.706
DPS	285	0.420	0.773	0.744	0.802	0.547	0.786
N5PS	218	0.660	0.726	0.763	0.688	0.453	0.791
C5PS	266	0.566	0.740	0.763	0.718	0.481	0.762
N5C5PS	224	0.568	0.752	0.822	0.682	0.510	0.764
N10PS	219	0.429	0.762	0.758	0.766	0.524	0.756
C10PS	248	0.444	0.749	0.770	0.728	0.499	0.769
N10C10PS	225	0.482	0.771	0.764	0.778	0.542	0.744
N15PS	394	0.478	0.783	0.679	0.866	0.559	0.737
C15PS	311	0.401	0.825	0.726	0.903	0.646	0.812
N15C15PS	394	0.424	0.796	0.670	0.896	0.587	0.776

**Figure 3:**
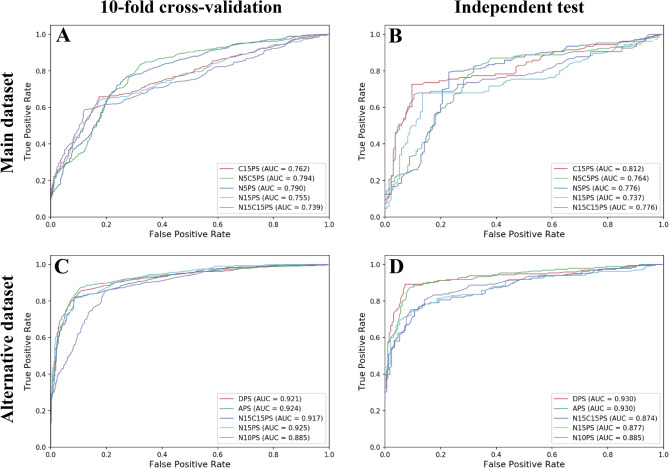
ROC curves of top-five types of propensity scores over tenfold cross-validation (**A**,**C**) and independent test (**B**,**D**) on main (**A**,**B**) and (**C**,**D**) alternative dataset.

As can be seen from Table 1 and Supplementary Table S1, the best Ac of 0.754 with an MCC of 0.496 and AUC of 0.762 was achieved by using C15PS (Fig. 3A). Meanwhile, the use of N5C5PS and N5PS performed well with correspondingly second and third highest Ac/MCC of 0.750/0.508 and 0.750/0.504, respectively. As noticed in Table 1, the performance of the widely used DPS (affording an Ac of 0.726 and AUC of 0.754) was comparable to that of the C15PS with regards to all of the five evaluation indices. In the case of independent test results, Table 2 showed that the C15PS also achieved better performance than other types of propensity scores and provided an Ac of 0.825 and an MCC of 0.646 (Fig. 3B). In the meanwhile, N15C15PS and N15PS performed well with the second and third highest independent test with Ac of 0.796 and 0.783, respectively. Hence, we selected the FSCM-based classifier in conjunction with propensity scores of 400 dipeptides on the C15 terminus (C15PS) as the optimal classifier for ACP identification using the main dataset. These results implied that the local sequential information plays a crucial role in distinguishing ACPs from non-ACPs than that of the global sequential information.

### Performance evaluation on alternative dataset

In this section, the same experimental setting as those used in the main dataset (from the original work from which it was taken) was utilized to determine which types of propensity scores were the most effective for distinguishing ACPs from random peptides in the alternative dataset. A series of performance comparison experiments using various types of propensity scores was carried out and their results were compared via a tenfold cross-validation and independent test as summarized in Tables 3 and 4.

**Table 3:** Cross-validation results of FSCM models with various types of sequence features as evaluated on the alternative dataset.

Method	Threshold	Fitness score	Ac	Sn	Sp	MCC	AUC
APS	418	0.942	0.884	0.870	0.898	0.770	0.924
DPS	198	0.626	0.872	0.852	0.893	0.746	0.921
N5PS	178	0.910	0.823	0.806	0.841	0.649	0.873
C5PS	111	0.947	0.786	0.836	0.736	0.576	0.858
N5C5PS	201	0.943	0.819	0.794	0.845	0.640	0.880
N10PS	199	0.840	0.834	0.858	0.809	0.669	0.885
C10PS	171	0.859	0.795	0.789	0.802	0.592	0.863
N10C10PS	195	0.860	0.831	0.855	0.805	0.662	0.890
N15PS	228	0.853	0.865	0.807	0.912	0.727	0.925
C15PS	161	0.821	0.798	0.777	0.818	0.596	0.864
N15C15PS	234	0.871	0.867	0.815	0.909	0.731	0.917

**Table 4:** Independent test results of FSCM models with various types of sequence features as evaluated on the alternative dataset.

Method	Threshold	Fitness score	Ac	Sn	Sp	MCC	AUC
APS	418	0.942	0.889	0.876	0.902	0.779	0.930
DPS	198	0.626	0.910	0.892	0.928	0.820	0.930
N5PS	178	0.910	0.796	0.756	0.838	0.596	0.867
C5PS	111	0.947	0.725	0.756	0.692	0.450	0.807
N5C5PS	201	0.943	0.791	0.751	0.832	0.585	0.858
N10PS	199	0.840	0.829	0.812	0.847	0.658	0.885
C10PS	171	0.859	0.787	0.812	0.759	0.572	0.854
N10C10PS	195	0.860	0.829	0.828	0.829	0.657	0.876
N15PS	228	0.853	0.824	0.769	0.873	0.647	0.877
C15PS	161	0.821	0.807	0.778	0.835	0.614	0.864
N15C15PS	234	0.871	0.827	0.769	0.880	0.655	0.874

From Table 3, it could be seen that the model affording the highest Ac had a value of 0.884 with a corresponding MCC of 0.770 and an AUC of 0.924 that was achieved using APS (Fig. 3C), while models affording the second and third highest Ac had values of 0.872 and 0.867, respectively, which were obtained using DPS and N15C15PS, respectively. As for results from the independent test (Table 2), both APS and DPS were amongst the 2 top-ranked classifiers also having the highest prediction results. Furthermore, it was found that DPS achieved slightly better performances than APS (0.910 vs 0.889 for Ac and 0.820 vs 0.779 for MCC). In the meanwhile, APS was found to achieve very comparable than that of the DPS feature as deduced from the AUC value (Fig. 3D). Hence, we selected the FSCM-based classifier in conjunction with the propensity scores of 20 amino acids from the whole sequence (APS) as the optimal classifier for ACP identification on alternative dataset. For convenience, the FSCM method in conjunction with the selected propensity scores (C15PS and APS for main and alternative datasets, respectively) will be referred to as the iACP-FSCM. Based on the observations described above, it could be demonstrated that the iACP-FSCM could provide the satisfied results for both main and alternative datasets because the composition information on ACPs influenced the interaction on cancer cell membrane, penetration the cell membrane, and then cancer cell cytotoxicity via their physicochemical properties (e.g. amphipathicity, hydrophobicity, and secondary structures)^1^.

### Comparisons of iACP-FSCM with existing methods

To further assess the predictive efficiency and effectiveness of the proposed iACP-FSCM, we compared its performances against existing methods on the same benchmark dataset. Table 5 lists performance comparisons of iACP-FSCM with existing methods on main and alternative datasets over independent test. The prediction results of existing methods (i.e. AntiCP^8^, iACP^10^, ACPred^19^, PEPred-Suite^20^, ACPred-FL^15^, ACPred-Fuse^18^ and AntiCP_2.0^25^) recorded in Table 5 come directly from the work^25^.

**Table 5:** Independent test results of the proposed method ACPred-FSCM with state-of-the-art methods as evaluated on main and alternative datasets.

Methods^a^	Main dataset				Alternative dataset			
	Ac	Sn	Sp	MCC	Ac	Sn	Sp	MCC
AntiCP	0.506	1.000	0.012	0.070	0.900	0.897	0.902	0.800
iACP	0.551	0.779	0.322	0.110	0.776	0.784	0.768	0.550
ACPred	0.535	0.856	0.214	0.090	0.853	0.871	0.835	0.710
PEPred-Suite	0.535	0.331	0.738	0.080	0.575	0.402	0.747	0.160
ACPred-FL	0.448	0.671	0.225	-0.120	0.438	0.602	0.256	-0.150
ACPred-Fuse	0.689	0.692	0.686	0.380	0.789	0.644	0.933	0.600
AntiCP_2.0	0.754	0.775	0.734	0.510	0.920	0.923	0.918	0.840
iACP-FSCM	0.825	0.726	0.903	0.646	0.889	0.876	0.902	0.779

^a^Results obtained from the published findings of AntiCP_2.0^25^.

By observing the results listed in Table 5, it is clearly that the performance of iACP-FSCM is superior to that of existing methods with the highest Ac (0.825), Sp (0.903) and MCC (0.646). Improvements of 7%, 17% and 14% for Ac, Sp and MCC on main dataset, respectively, were observed when compared with the state-of-the-art method AntiCP_2.0. In addition, iACP-FSCM achieved a greater than 14% increase in Ac compared with the existing ensemble methods containing PEPred-Suite, ACPred-FL and ACPred-Fuse. Although, AntiCP and ACPred were higher Sn values than the proposed iACP-FSCM, the corresponding Sp and MCC were significantly lower. In case of the comparative results on alternative dataset, we noticed that AntiCP_2.0 provided the highest accuracy of 0.920 with an MCC of 0.840 (Table S3). Meanwhile, the second- and third-best ACP predictors (Ac, MCC) were obtained from AntiCP (0.900, 0.800) and iACP-FSCM (0.889, 0.779), respectively. Although, AntiCP_2.0 obtained better prediction results than our proposed iACP-FSCM, AntiCP_2.0 is limited in terms of interpretability and practical utility for biologists and biochemists. On the other hand, the iACP-FSCM provides the propensity scores that might provide the crucial information relating to local and global properties of ACPs, which is easily understood and implemented. Furthermore, the interpretability of the proposed iACP-FSCM with impressive prediction performance is a more useful and practical approach. Taken together, these results revealed that iACP-FSCM provided more impressive prediction performances on both main and alternative datasets in terms of simplicity, interpretability and generalizability.

### Characterization of anticancer activities of peptides using propensity scores

Unlike black-box modeling methods such as SVM and ensemble methods, the advantage of iACP-FSCM are that the estimated propensity scores of amino acids and dipeptides derived from the FSCM method could easily identify informative PCPs for gaining a more in-depth understanding on the characteristics of anticancer activities peptides. The propensity scores of 20 amino acids to be ACPs derived from the DPS (Fig. 2) are recorded in Table 6, which were calculated using Matlab (R2020a). The five amino acids with the highest propensity scores contained Tyr, Trp, His, Met and Lys (355.55, 328.60, 317.03, 311.58 and 296.78, respectively), whereas the five amino acids with the lowest propensity scores contained Gln, Val, Gly, Cys and Arg (198.45, 212.55, 225.08, 226.38 and 229.63, respectively). In case of the propensity scores of 400 dipeptides to be ACPs, Fig. 2 shows that the five top-ranked dipeptides with the highest propensity scores contained KK, LW, GH, HI and MY, whereas the five top-ranked dipeptides with the lowest propensity scores contained KG, LD, LV, CR and TT.

**Table 6:** Important physicochemical properties (PCPs) as derived from the iACP-FSCM.

Amino acid	PS- DPPVI (rank)	MITS020101^a^ (rank)	QIAN880113^b^ (rank)	JOND750101^c^ (rank)
Y-Tyr	355.55 (1)	5.06 (2)	0 (9)	2.67 (5)
W-Trp	328.60 (2)	6.93 (1)	0.36 (2)	3.77 (1)
H-His	317.03 (3)	1.45 (5)	0.29 (3)	0.87 (12)
M-Met	311.58 (4)	0 (15)	0.11 (5)	1.67 (8)
K-Lys	296.78 (5)	3.67 (3)	0.45 (1)	1.64 (9)
A-Ala	295.15 (6)	0 (8)	− 0.08 (11)	0.87 (11)
L-Leu	288.23 (7)	0 (14)	0.28 (4)	2.17 (6)
P-Pro	276.55 (8)	0 (17)	− 0.42 (20)	2.77 (4)
E-Glu	272.83 (9)	1.27 (6)	− 0.19 (15)	0.67 (14)
S-Ser	268.65 (10)	0 (18)	0.07 (6)	0.07 (18)
I-Ile	247.03 (11)	0 (13)	− 0.01 (10)	3.15 (2)
D-Asp	244.80 (12)	0 (10)	− 0.24 (16)	0.66 (15)
F-Phe	243.43 (13)	0 (11)	0 (8)	2.87 (3)
T-Thr	242.05 (14)	0 (19)	− 0.33 (19)	0.07 (19)
N-Asn	232.70 (15)	0 (16)	− 0.08 (12)	0.09 (17)
R-Arg	229.63 (16)	2.45 (4)	0.05 (7)	0.85 (13)
C-Cys	226.38 (17)	0 (9)	− 0.25 (17)	1.52 (10)
G-Gly	225.08 (18)	0 (12)	− 0.1 (13)	0.1 (16)
V-Val	212.55 (19)	0 (20)	− 0.13 (14)	1.87 (7)
Q-Gln	198.45 (20)	1.25 (7)	− 0.28 (18)	0 (20)
Correlation (R)	1.000	0.577	0.569	0.541

^a^MITS020101 = Amphiphilicity index (Mitaku et al., 2002).

^b^QIAN880113 = Weights for alpha-helix at the window position of 6 (Qian-Sejnowski, 1988).

^c^JOND750101 = Hydrophobicity (Jones, 1975).

In biological process, cancer cell development is mostly caused by free radicals damaged on cells via ionizing radiation mechanism, especially DNA damage^5^. Meanwhile, reactive oxygen species can promote cancer, growth arrest, cytotoxicity and irreversible damage. The amino acid composition on ACPs can act as antioxidant and dietary source of the cells^4^. Interestingly, the five amino acids with the highest propensity scores were reported as the important factor for the antioxidant activity. Because electron-rich aromatic rings in side chains of Tyr and Trp, sulfur atoms with two lone electron pairs in side chains of Met, and nitrogen atoms with one lone electron in side chain of His are easily oxidized^41^. Among anti-oxidative amino acids, Trp is low abundant in natural peptides, but, it is crucial role of biomolecule activity and easy chemical modification^42^. Although, His is one of the five top-ranked amino acids, His-containing dipeptides such as GH and HI, had no anticancer activity in in vitro study. Furthermore, AH and LH showed antiangiogenic activity without great anticancer potential in zebrafish embryo model^43^.

It is well recognized that cancer metabolism has focused on glycolysis and tricarboxylic acid (TCA) cycle. Many cancer cells are highly dependent on Gln and Ser uptake for a proliferation and these two amino acids are the most highly consumed nutrients^44^. Choi and Coloff proposed that Gln serves as anaplerosis metabolite and plays a crucial role in the TCA cycle to maintain mitochondrial ATP production^45^. Meanwhile, the tumor’s evolution utilizes Gln, as alternative fuels to optimize a nutrient utilization. Similarly, Val, which is one of branched-chain amino acid, can fuel in the TCA cycle^46^. Gln and Gly, which provide essential carbon and nitrogen sources for the nucleobase synthesis, are beneficial in the energy-consuming process via DNA/RNA synthesis in cells^47^. Although, Gly is one of the five top-ranked amino acids having lowest propensity scores, dipeptide containing Gly or Pro performed good cytotoxicity in vitro tumor human cell lines such as A549 lung cancer cell line^48^. After analyzing the FSCM-derived propensity scores, these results suggest that amino acids having high propensity scores could be important in exhibiting the anticancer activity via the oxidation protection process, while amino acids having low propensity scores could be important in serving as dietary source of the cancer cells as well as provide a contradictory effect on anticancer activity.

### Characterization of anticancer activities using informative physicochemical properties

In this section, the iACP-FSCM method was utilized to provide a more in-depth understanding of the basis and important factors for the anticancer activity. In the previous studies, the physicochemical properties (i.e. amino acid sequence, length, net charge, secondary structure, amphipathicity, and hydrophobicity) of peptides play crucial role in their hemolytic activity, penetration ability and anticancer/antitumor activity^1,19,49–52^. The three importantly selected PCPs derived from iACP-FSCM consist of MITS020101 (Amphiphilicity index), QIAN88011 (Weights for alpha-helix at the window position of 6) and JOND750101 (Hydrophobicity) were showed in Table 6. In addition, Supplementary Table S4 presents further details of the top-twenty informative PCPs.

It is well-known that Trp with a propensity score of 328.20 is a common amino acid in amphiphilicity, alpha-helix, and hydrophobicity. Lee et al. investigated the relationship between the anticancer activities of Pep27 analogues and their hemolytic activity and hydrophobicity. They found that Pep27 analogue peptides substituting with Trp was increased hydrophobicity based on the RP-HPLC retention time. The substitutions of (^11^Ser → Trp) and (^13^Qln → Trp) in Pep27anal2 had the greatest hydrophobicity with a RP-HPLC retention time of 22.50 min as well as exhibited the most anticancer activity with the IC_50_ (10–28 μM) and IC_90_ (35–55 μM) in five cancer cell lines^41^. This observation was quite consistent with the previous work of^53,54^, implying that end-capping and cyclization of hexameric peptide sequences of RRWQWR and RRWWRF or end-tagging of short peptides KNK10 (KNKGKKNGKH) and GKH17 (GKHKNKGKKNGKHNGWK) with hydrophobic Trp or Phe stretches could enhance the stability of ACPs and against proteolytic degradation.

Table 6 shows that Lys, His and Arg (i.e. the cationic amino acids) provide acceptable propensity scores for both amphiphilicity index (MITS020101) and alpha-helix (QIAN88011) properties. These three amino acids are described by the amphipathic alpha-helical structure transformation that segregates Lys on one face and Ile on the opposite side to interact with the negatively-charged membrane that consequently gives rise to high anticancer activity^53,55^. Furthermore, the octahistidine-octaarginine (H_8_R_8_) peptide is a common cationic cell penetrating peptide with endosomal escape capabilities. The modified H_8_R_8_ as a lipid-modified cationic peptide (i.e. stearyl-H_8_R_8_ and vitamin E succinate-H_8_R_8_) with the functions of amphiphilic, biodegradable and lipid structure, can increase reactive oxygen species production, reduce cell bioenergetics and drug efflux, trigger apoptosis and G1 cell cycle arrest, and mitochondria depolarization thereby leading to cancer cell toxicity and death^56^. Owing to the fact that the indole side chains of Trp exhibits a preference to interact with the interfacial region of lipid bilayers while Lys and Arg side chains on peptides provide positive charges and hydrogen bonding capabilities to attract negatively-charged phospholipid headgroups of cell membranes^54,57,58^. Furthermore, side chains of aromatic residues (i.e. Trp and Phe) in which one side of the backbone ring forms a hydrophobic face to engage in interaction with the micelle^6^. Such interaction between ACPs containing Trp, Phe, Lys, His, or Arg and cancer cell membranes are often found in situations of cancer cell eradication. The aforementioned results as obtained from iACP-FSCM are in accordance with previous studies^6,53–59^ in which physicochemical properties of ACPs (i.e. amphiphilicity, helical structure and hydrophobicity) pertains to the interaction between ACPs and the cell surface. This interaction causes ACPs to transform into a helical structure to confer the spatial arrangement of aliphatic side chains for membrane insertion. The turn stabilization of the helical conformation promotes the intra-chain hydrogen-bonding and mediates the backbone hydrophobicity thereby causing a deeper insertion of peptides into the lipid bilayer^59^.

### Case study

A key advantage of iACP-FSCM is its interpretability to biologists in which mechanistic insights into the origin of anticancer activity of investigated ACPs as deduced from the scoring function S(P) for ACPs that have not yet been experimentally verified^26,27,37^. The top 20 peptides with the highest and lowest scores are reported in Supplementary Tables S5 and S6, respectively. We noticed that scores for the top 20 ACPs with the highest ACP scores (S(P)) were in ranges of 636.59–700.64 whereby the threshold value was 311 (Table 1). Interestingly, the peptide sequence of KAKLF having an ACP score of 645 was found in the top 9 peptides having a high docking score of -29.75 kJ/mol towards the hypoxia inducible factor 1α (HIF-1α) as reported in the previous study^60^.

Inspired by this study^60^, the top 20 ACPs (ID: 1–20) derived from the iACP-FSCM were then docked with the predicted binding sites of HIF-1α in order to estimate their interaction energies (kcal/mol) for finding a new potential peptide-based drug for HIF-1α. In order to make a fair comparison, the same experimental setup was used for estimating interaction energies of the top 9 ACPs as proposed by the previous study^60^. In this study, HIF-1α was prepared for docking using the protein preparation features in the Chimera software, which was performed using the default protocol for PDB2PQR and Dock Prep. Protonation states were assigned using PROPKA at a pH of 7.0 and Gasteiger charges were assigned to the protein^61^. Protein-peptide similarity-based docking was performed using the GalaxyPepDock web server (http://galaxy.seoklab.org/pepdock) by utilizing the information provided by the database to perform the docking procedure that entails the search for suitable templates from a database of experimentally determined structures and building models using the energy-based optimization method that allows for structural flexibility. The calculation of protein-peptide binding and interaction energy were performed using the NOVA force field^62^ while the visualization of the structures was carried out using YASARA (Yet Another Scientific Artificial Reality Application; http://www.yasara.org/index.html) .

**Figure 4:**
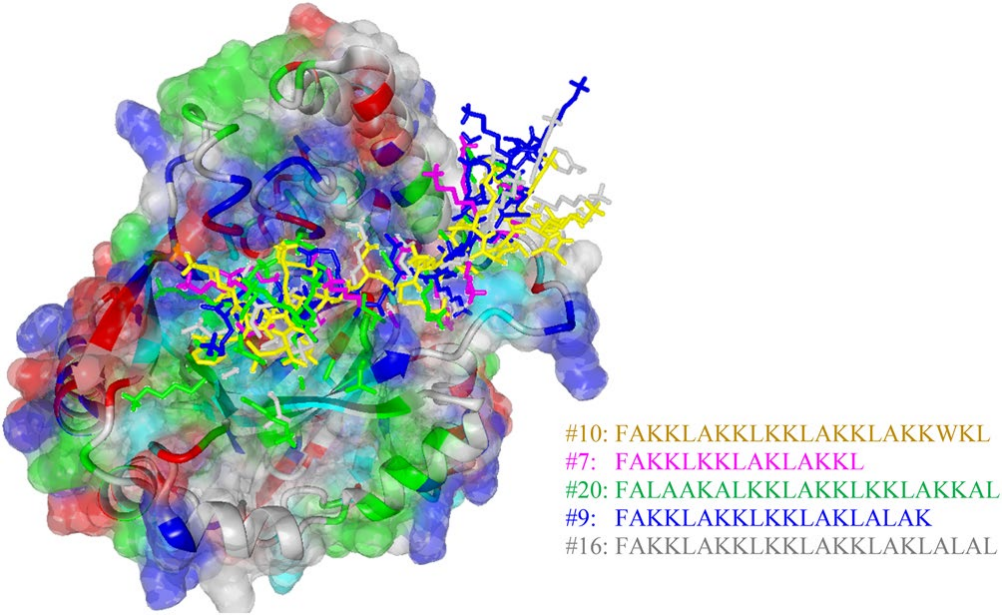
Three-dimensional complex structure of the top 5 ACPs having maximum interaction energies**.** The binding pocket was colored according to residue type by YASARA coloring scheme, where grey, green, blue, red and cyan colors represent non-polar, amidic, basic, acidic hydroxylic amino acids, respectively.

**Figure 5:**
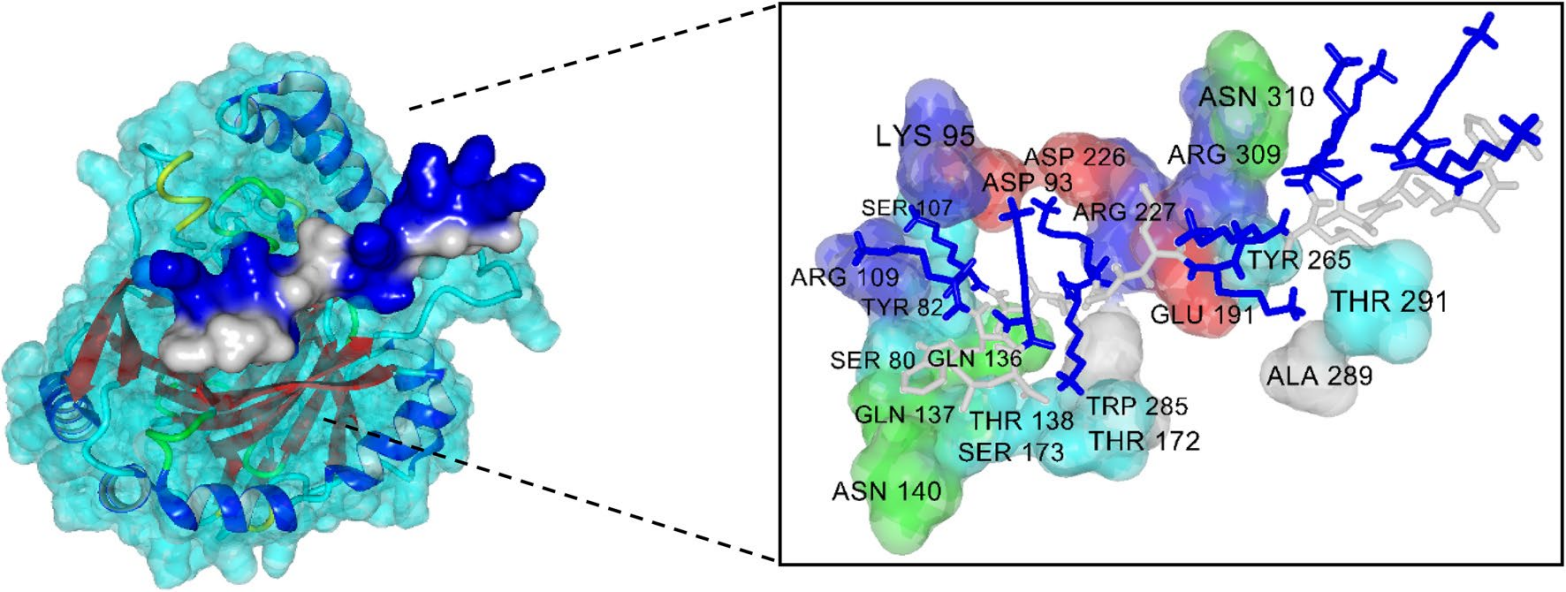
Molecular surface of docking complex between the HIF-1α receptor (left) and the peptide ID: 10 (right), in stick model, where amino acids in 3 Å binding, where Phe, Ala, Leu and Trp are non-polar residues (grey) and Lysis basic residue (blue).

The three-dimensional complexed structure for the top 5 potential ACPs is provided in Fig. 4 while the interaction energy scores are listed in Table 7 and it was found that values ranged from -9.39 kcal/mol to -6.53 kcal/mol (i.e. consisting of peptides ID 10, 7, 20, 9 and 16). Particularly, the peptide sequence, ACP score and their corresponding interaction energy (i.e. as reported in parenthesis) for peptides ID 10, 7, 20, 9 and 16 are as follows: (FAKKLAKKLKKLAKKLAKKWKL, 655.29, − 9.39), (FAKKLKKLAKLAKKL, 663.93, − 8.71), (FALAAKALKKLAKKLKKLAKKAL, 636.59, − 7.21), (FAKKLAKKLKKLAKLALAK, 657.22, − 6.73) and (FAKKLAKKLKKLAKKLAKLALAL, 646.64, − 6.53), respectively. A visualization of the molecular surface of peptide ID: 10 (peptide sequence FAKKLAKKLKKLAKKLAKKWKL) that was found to exhibit maximal interaction energy of − 9.39 kcal/mol (i.e. and within 3 Å distance) with the HIF-1α receptor is depicted in Fig. 5. As seen from Table 5, the interaction energies of ACPs ID: 21–29 are ranging from − 4.81 kcal/mol to 11.98 kcal/mol. Amongst the 9 ACPs as reported by a previous study^60^, peptide ID: 25 (i.e. having a peptide sequence KAKLF) displayed the highest interaction energy score of − 4.81 kcal/mol with the HIF-1α receptor. These results indicated that peptide ID: 10 as derived from this study is a promising ACP with promising potential against breast cancer when compared to peptide ID: 10 as proposed by the previous study^60^. However, additional in vitro and in vivo approaches will be needed for further development of novel ACPs against breast cancer. It is highly anticipated that iACP-FSCM can serve as an important tool for the rapid screening of promising ACPs against breast cancer as well as other types of cancer cell prior to their synthesis.

**Table 7:** Top 20 ACPs having high score derived from iACP-FSCM and top 9 ACPs having maximum docking scores derived from the work^53^ along with their ACP scores and interaction energies.

ID	Peptide sequence	ACP Score^c^	Interaction Energy ^d^
1	FALAKKALKKAKKAL^a^	700.64	− 3.69
2	FAKKLAKKLKKLAKKLAK^a^	692.71	3.94
3	FAKKLAKKLAKAL^a^	682.17	2.10
4	FAKKLAKKLKKLAKKLAKLAKKL^a^	679.14	− 1.95
5	FAKKLAKLAKKL^a^	673.36	− 2.04
6	FAKKLAKLAKKLAKAL^a^	667.27	0.21
7	FAKKLKKLAKLAKKL^a^	663.93	− 8.71
8	FAKKLAKLAKKALAL^a^	660.00	− 2.58
9	FAKKLAKKLKKLAKLALAK^a^	657.22	− 6.73
10	FAKKLAKKLKKLAKKLAKKWKL^a^	655.29	− 9.39
11	FAKLWAKLAKKL^a^	653.91	− 5.58
12	FALAKLAKKAKAKLKKALKAL^a^	653.40	− 2.58
13	FAKKLAKKLAKLL^a^	652.75	1.59
14	FAKKLAKKLAKLAL^a^	650.85	− 2.75
15	FAKLLAKLAKK^a^	649.10	− 4.52
16	FAKKLAKKLKKLAKKLAKLALAL^a^	646.64	− 6.53
17	KAKLF^a^	645.00	− 4.81
18	FAKKALKALKKL^a^	645.00	0.00
19	FAKKLAKLAKKLAKLAL^a^	642.75	− 4.92
20	FALAAKALKKLAKKLKKLAKKAL^a^	636.59	− 7.21
21	FALALKA^b^	530.50	− 2.96
22	RYLGYL^b^	314.40	− 2.01
23	FALA^b^	N/A	0.17
24	FAKLA^b^	571.25	3.29
25	KAKLF^b^	645.00	− 4.81
26	ERRP^b^	269.00	11.98
27	WALAL^b^	473.75	− 1.69
28	MTLTG^b^	349.75	− 0.44
31	KWKLF^b^	N/A	− 4.60

^a^The peptide sequences are the top 20 ACPs having high score derived from iACP-FSCM derived from this study.

^b^The peptide sequences are the top 9 ACPs having maximum docking interactions scores derived from the work^53^.

^c^ACP scores are calculated using the scoring function S(P) and N/A means that the peptide is not found in the benchmark dataset.

^d^Interaction Energy is Interaction Energy Yasara Nova Force Field (kcal/mol).

## Conclusions

In this study, we have proposed for the first time a computational model called the iACP-FSCM for ACP identification and characterization via the use of propensity scores of local and global sequential information as obtained using the novel FSCM method. It was demonstrated that the iACP-FSCM could easily identify ACPs using only a weighted-sum score and a single threshold value. This was compared with the complex ensemble classifiers as developed using a large number of ML classifiers and feature descriptor schemes. Furthermore, the FSCM-derived propensity scores can be adopted to identify informative physicochemical properties that might provide crucial information relating to local and global properties of ACPs. Results from the benchmarked comparison validated the effectiveness and robustness of the proposed iACP-FSCM approach. We further applied the iACP-FSCM to identify potential peptide-based drugs against HIF-1α and obtained a list of potential peptides against HIF-1α. With these promising results, it is highly anticipated that iACP-FSCM can serve as an important tool for the rapid screening of promising ACPs against various types of cancer cells prior to their synthesis. In order to develop a convenient bioinformatics tool, the proposed model is deployed as a web server that is made publicly available at http://camt.pythonanywhere.com/iACP-FSCM. Owing to the high potential of the FSCM method as proposed in this study, the method could be easily applied for predicting and characterizing other therapeutic peptides without any major modifications, such as cell-penetrating peptides^63^, antiviral peptides^20,23^ and predicting antihypertensive^20,23^, hemolytic peptide^31^.

## Supplementary Information

Supplementary Information
