# Supplementary material for: Improved prediction and characterization of anticancer activities of peptides using a novel flexible scoring card method
Source: Sci Rep. 2021 Feb 4;11:3017. doi: 10.1038/s41598-021-82513-9 (PMC7862624; doi:10.1038/s41598-021-82513-9)
Supplement: Supplementary file 1 — Supplementary Information [file 41598_2021_82513_MOESM1_ESM.pdf]

Improved prediction and characterization of anticancer activities of peptides using a novel flexible scoring card method

Phasit Charoenkwan[1], Wararat Chiangjong[2], Vannajan Sanghiran Lee[3], Chanin Nantasenamat[4], Md. Mehedi Hasan[5], Watshara Shoombuatong[4,*]

[1]Modern Management and Information Technology, College of Arts, Media and Technology, Chiang Mai University, Chiang Mai, Thailand, 50200
[2]Pediatric Translational Research Unit, Department of Pediatrics, Faculty of Medicine, Ramathibodi Hospital, Mahidol University, Bangkok 10400, Thailand
[3]Department of Chemistry, Centre of Theoretical and Computational Physics, Faculty of Science, University of Malaya, Kuala Lumpur, 50603 Malaysia
[4]Center of Data Mining and Biomedical Informatics, Faculty of Medical Technology, Mahidol University, Bangkok, Thailand, 10700.
[5]Department of Bioscience and Bioinformatics, Kyushu Institute of Technology, 680-4 Kawazu, Iizuka, Fukuoka 820-8502, Japan.

*Corresponding author: watshara.sho@mahidol.ac.th

Performance evaluation

In order to evaluate the prediction ability of the model, four widely used metrics in binary classification were employed as follows:

$$\mathrm{Ac} = \frac{\mathrm{TP} + \mathrm{TN}}{(\mathrm{TP} + \mathrm{TN} + \mathrm{FP} + \mathrm{FN})}$$

$$\mathrm{Sn} = \frac{\mathrm{TP}}{(\mathrm{TP} + \mathrm{FN})}$$

$$\mathrm{Sp} = \frac{\mathrm{TN}}{(\mathrm{TN} + \mathrm{FP})}$$

$$\mathrm{MCC} = \frac{\mathrm{TP} \times \mathrm{TN} - \mathrm{FP} \times \mathrm{FN}}{\sqrt{(\mathrm{TP} + \mathrm{FP})(\mathrm{TP} + \mathrm{FN})(\mathrm{TN} + \mathrm{FP})(\mathrm{TN} + \mathrm{FN})}}$$

where Ac, Sn, Sp and MCC represents the accuracy, sensitivity, specificity and Matthews coefficient correlation, respectively. TP represents the number of correctly predicted peptides in the positive dataset (ACPs), TN denotes the number of correctly predicted peptides in the negative dataset (non- ACPs), FP represents the number of incorrectly predicted peptides in the positive dataset and FN represents the number of incorrectly predicted peptides in the negative dataset. Model comparison of the proposed model when those of previously described models was performed via the use of the receiver operating characteristic (ROC) curve of threshold-independent parameters. Correspondingly the area under the ROC curve was utilized to assess the prediction performance whereby AUC values in the range of 0.5 and 1 are indicative of random and perfect models, respectively.

Genetic algorithm

The GA algorithm used in the FSCM for optimizing init-C15PS consists of the following steps:
Step 1: (Initialization) Randomly generate 40 sets of C15PS that includes the init-C15PS.
Step 2: (Evaluation) Compute fitness values from the fitness function (Eq.1) for all sets of C15PS in order to determine the best C15PS in the population.
Step 3: (Crossover) Perform a 20-point crossover between the best C15PS and other C15PS.
Step 4: (Mutation) Randomly mutate individuals (except the best C15PS) using the mutation probability of Pm = 0.01 with a real-valued mutation operator.
Step 5: (Termination) Stop the GA algorithm if the termination condition is reached, otherwise proceed to Step 2. In this study, 20 generations were used as the stop condition.
More details concerning the optimization procedure of dipeptides propensity scores using the GA algorithm can be found in our previous studies [1, 2].

Step-by-step instructions for using the iACP-FSCM web server

The iACP-FSCM webserver is used to predict whether a given peptide is ACP or not. We have provided 2 prediction models (i.e. C15PS and DPS) for ACP prediction. The C15PS model is used to predict a peptide having more than and equal to 15 amino acids while the DPS model is used to predict all peptides without the length condition.

Step 1: Proceed to the web server at http://camt.pythonanywhere.com/iACP-FSCM.

Step 2: Copy/paste the query protein sequences into the text box or upload a FASTA file by clicking on the “Choose file” button. Examples of FASTA-formatted sequences can be seen by clicking on the “example file” link found below the input box.

Step 3: Click on the “Submit” button in order to perform the prediction.

Step 4: Prediction results and their corresponding their ACP scores are outputted as shown on the right-hand panel of the web server. Moreover, we will regularly maintain and keep the iACP-FSCM web server up-to-date so as to provide optimal performance to the scientific community.

Table S1. Cross-validation and independent test results of FSCM models with propensity scores of the DPC for 15 residues present at C terminal region (C15PS) as evaluated on the main dataset

#Exp	Threshold	Fitness score	10-fold CV		Independent test			
			**Ac**	**MCC**	**Ac**	**Sn**	**Sp**	**MCC**
1	293	0.365	0.742	0.473	0.817	0.698	0.910	0.630
2	332	0.412	0.740	0.469	0.817	0.736	0.881	0.627
3	280	0.387	0.742	0.472	0.821	0.774	0.858	0.635
4	332	0.366	0.748	0.485	0.775	0.726	0.813	0.542
**5**	**311**	**0.401**	**0.754**	**0.496**	**0.825**	**0.726**	**0.903**	**0.646**
6	304	0.364	0.740	0.466	0.825	0.717	0.910	0.647
7	312	0.385	0.744	0.477	0.779	0.651	0.881	0.552
8	298	0.402	0.739	0.466	0.808	0.717	0.881	0.610
9	324	0.397	0.745	0.480	0.829	0.745	0.896	0.653
10	292	0.396	0.751	0.491	0.813	0.783	0.836	0.619
Mean	307.800	0.388	0.744	0.477	0.811	0.727	0.877	0.616
STD.	17.681	0.017	0.005	0.010	0.019	0.037	0.032	0.039

The experiment #5 having the optimal prediction performances over both 10-fold CV and independent test is used for further analysis.

Table S2. Cross-validation and independent test results of FSCM models with propensity scores of amino acids (APS) as evaluated on the alternative dataset

#Exp	Threshold	Fitness score	10-fold CV		Independent test			
			Ac	MCC	Ac	Sn	Sp	MCC
1	399	0.931	0.879	0.760	0.881	0.871	0.892	0.763
2	384	0.925	0.884	0.770	0.887	0.861	0.912	0.774
3	387	0.947	0.878	0.758	0.881	0.866	0.897	0.763
4	461	0.948	0.876	0.755	0.876	0.856	0.897	0.753
**5**	**418**	**0.942**	**0.884**	**0.770**	**0.889**	**0.876**	**0.902**	**0.779**
6	412	0.940	0.884	0.770	0.881	0.871	0.892	0.763
7	403	0.944	0.882	0.766	0.881	0.871	0.892	0.763
8	436	0.955	0.875	0.753	0.884	0.851	0.918	0.770
9	461	0.947	0.874	0.751	0.881	0.871	0.892	0.763
10	388	0.945	0.882	0.767	0.889	0.866	0.912	0.779
Mean	414.900	0.942	0.880	0.762	0.883	0.866	0.901	0.767
STD.	28.992	0.009	0.004	0.008	0.004	0.008	0.010	0.008

The experiment #5 having the optimal prediction performances over both 10-fold CV and independent test is used for further analysis.

Table S3. Cross-validation and independent test results of ACPred-FSCM and AntiCP_2.0 as evaluated on main and alternative datasets

Dataset	Cross-validation	Methods	Ac	Sn	Sp	MCC	AUC
Main	10-fold CV	AntiCP_2.0	0.753	0.741	0.765	0.510	0.830
		iACP-FSCM	0.754	0.656	0.829	0.496	0.762
	Independent test	AntiCP_2.0	0.754	0.775	0.734	0.510	0.830
		iACP-FSCM	0.825	0.726	0.903	0.646	0.812
Alternative	10-fold CV	AntiCP_2.0	0.901	0.902	0.900	0.800	0.970
		iACP-FSCM	0.872	0.852	0.893	0.746	0.921
	Independent test	AntiCP_2.0	0.920	0.923	0.918	0.840	0.970
		iACP-FSCM	0.910	0.892	0.928	0.820	0.930

Table S4. The twenty top-ranked informative physicohemical properties having the highest pearson correlation (R) with the propensity scores of amino acids

Rank	AAindex	R	Description
1	MITS020101	0.577	Amphiphilicity index (Mitaku et al., 2002)
2	QIAN880113	0.569	Weights for alpha-helix at the window position of 6 (Qian-Sejnowski, 1988)
3	RICJ880110	0.552	Relative preference value at C5 (Richardson-Richardson, 1988)
4	SNEP660103	0.548	Principal component III (Sneath, 1966)
5	HUTJ700101	0.546	Heat capacity (Hutchens, 1970)
6	AURR980118	0.522	Normalized positional residue frequency at helix termini C" (Aurora-Rose, 1998)
7	SIMZ760101	0.511	Transfer free energy (Simon, 1976), Cited by Charton-Charton (1982)
8	FASG760102	0.509	Melting point (Fasman, 1976)
9	TAKK010101	0.501	Side-chain contribution to protein stability (kJ/mol) (Takano-Yutani, 2001)
10	FAUJ880106	0.485	STERIMOL maximum width of the side chain (Fauchere et al., 1988)
11	RACS820105	0.480	Average relative fractional occurrence in E0(i) (Rackovsky-Scheraga, 1982)
12	LEVM760107	0.470	van der Waals parameter epsilon (Levitt, 1976)
13	FINA770101	0.462	Helix-coil equilibrium constant (Finkelstein-Ptitsyn, 1977)
14	HARY940101	0.456	Mean volumes of residues buried in protein interiors (Harpaz et al., 1994)
15	QIAN880112	0.453	Weights for alpha-helix at the window position of 5 (Qian-Sejnowski, 1988)
16	CHAM820101	0.452	Polarizability parameter (Charton-Charton, 1982)
17	JOND750101	0.451	Hydrophobicity (Jones, 1975)
18	ARGP820101	0.450	Hydrophobicity index (Argos et al., 1982)
19	NOZY710101	0.449	Transfer energy, organic solvent/water (Nozaki-Tanford, 1971)
20	FAUJ880103	0.449	Normalized van der Waals volume (Fauchere et al., 1988)

Table S5. The 20 highest-ranked peptides using a novel flexible scoring card method

#	Peptide sequence	Score	PP1	PP2	PP3	PP4	PP5	PP6	PP7	PP8
1	FALAKKALKKAKKAL	700.64	-0.2	0.56	0.56	-0.01	1.38	0.48	6	helix-coil
2	FAKKLAKKLKKLAKKLAK	692.71	-0.32	0.58	0.58	-0.52	1.74	0.81	9	helix
3	FAKKLAKKLAKAL	682.17	-0.16	0.56	0.56	0.14	1.31	0.36	5	helix-coil
4	FAKKLAKKLKKLAKKLAKLAKKL	679.14	-0.29	0.58	0.58	-0.35	1.68	0.72	11	helix-coil
5	FAKKLAKLAKKL	673.36	-0.2	0.56	0.56	0.01	1.41	0.43	5	helix-coil
6	FAKKLAKLAKKLAKAL	667.27	-0.15	0.56	0.56	0.21	1.3	0.34	6	helix-coil
7	FAKKLKKLAKLAKKL	663.93	-0.26	0.57	0.57	-0.24	1.61	0.61	7	helix-coil
8	FAKKLAKLAKKALAL	660.00	-0.1	0.55	0.55	0.47	1.15	0.18	5	helix-coil
9	FAKKLAKKLKKLAKLALAK	657.22	-0.21	0.57	0.57	-0.02	1.47	0.5	8	helix
10	FAKKLAKKLKKLAKKLAKKWKL	655.29	-0.32	0.58	0.58	-0.64	1.76	0.7	11	helix
11	FAKLWAKLAKKL	653.91	-0.08	0.54	0.54	0.24	1.13	-0.06	4	coil-helix-coil
12	FALAKLAKKAKAKLKKALKAL	653.40	-0.17	0.56	0.56	0.15	1.33	0.41	8	helix
13	FAKKLAKKLAKLL	652.75	-0.14	0.56	0.56	0.28	1.31	0.27	5	helix-coil
14	FAKKLAKKLAKLAL	650.85	-0.12	0.55	0.55	0.38	1.22	0.22	5	helix
15	FAKLLAKLAKK	649.10	-0.12	0.55	0.55	0.33	1.22	0.22	4	helix
16	FAKKLAKKLKKLAKKLAKLALAL	646.64	-0.17	0.57	0.57	0.21	1.38	0.37	9	helix-coil
17	KAKLF	645.00	-0.14	0.52	0.52	0.1	1.22	0.2	2	coil
18	FAKKALKALKKL	645.00	-0.2	0.56	0.56	0.01	1.41	0.43	5	helix-coil
19	FAKKLAKLAKKLAKLAL	642.75	-0.12	0.56	0.56	0.41	1.22	0.22	6	helix
20	FALAAKALKKLAKKLKKLAKKAL	636.59	-0.19	0.59	0.59	0.13	1.44	0.44	9	helix-coil

PP1 = Hydrophobicit
PP2 = Steric hindrance
PP3 = Sidebulk
PP4 = Hydropathicity
PP5 = Amphipathicity
PP6 = Hydrophilicity
PP7 = Charge
PP8 = Strucsture (PEP-FOLD3

Table S6. The 20 lowest-ranked peptides using a novel flexible scoring card method

#	Peptide sequence	Score	PP1	PP2	PP3	PP4	PP5	PP6	PP7	PP8
1	VSGNVAARKGKQQTSSGKGGGTN	128.14	-0.27	0.64	0.64	-1.06	0.69	0.39	4	coil-helix-coil
2	GLLSGILGAGKNIVCGLSGLC	136.05	0.18	0.62	0.62	1.31	0.17	-0.61	1	helix-coil
3	GICRCLCRRGVCRCICVL	138.29	-0.16	0.65	0.65	1.18	0.54	-0.23	4	helix-coil
4	RCRFCCRCCPRMRGCGICCRF	141.85	-0.37	0.65	0.65	0.12	0.7	0.09	6	helix-coil
5	GICRCICTRGFCRCICVL	142.47	-0.06	0.65	0.65	1.35	0.41	-0.48	3	sheet-coil-sheet
6	GFCRCLCRRGVCRCICTR	144.18	-0.33	0.65	0.65	0.35	0.68	0.06	5	helix-coil
7	PGLGFY	144.40	0.24	0.61	0.61	0.48	0	-1.1	0	coil
8	KQFRIRVRVIRK	146.82	-0.57	0.69	0.69	-0.76	1.53	0.76	6	coil
9	DLDVNVFNR	148.50	-0.25	0.71	0.71	-0.39	0.27	0.23	-1	helix-coil
10	TVYTNA	152.40	-0.03	0.62	0.62	-0.03	0	-0.82	0	coil
11	GIKCRFCCGCCTPGICGVCCRF	153.57	-0.03	0.64	0.64	1	0.39	-0.43	3	sheet-coil-sheet
12	VRPYLVAF	154.71	0.08	0.61	0.61	1.17	0.31	-0.89	1	helix
13	DTTFCRCRVSCNILEKYSGKCE LSGRTARICC	155.42	-0.27	0.63	0.63	-0.19	0.61	0.22	3	coil-helix-coil
14	VQLRIRVRVIRK	155.55	-0.44	0.68	0.68	0	1.23	0.44	5	coil
15	SMSGFSKPHD	157.56	-0.2	0.55	0.55	-1.03	0.51	0.26	0.5	coil
16	RCICTRGFC	157.63	-0.23	0.65	0.65	0.52	0.54	-0.19	2	coil
17	TTPLCVGVIIGLTTSIKICK	159.16	0.11	0.61	0.61	1.26	0.37	-0.56	2	coil
18	DLPECCSATELELDSGKQTS	162.00	-0.21	0.6	0.6	-0.62	0.44	0.52	-4	helix-coil
19	FIGAILPAIAGLVGGLINR	164.06	0.24	0.62	0.62	1.61	0.13	-0.78	1	helix-coil
20	IIGPVLGLVGKPLESLLE	166.59	0.16	0.6	0.6	1.13	0.34	-0.35	-1	helix-coil

PP1 = Hydrophobicit
PP2 = Steric hindrance
PP3 = Sidebulk
PP4 = Hydropathicity
PP5 = Amphipathicity
PP6 = Hydrophilicity
PP7 = Charge
PP8 = Strucsture (PEP-FOLD3)

Reference

[1] P. Charoenkwan, W. Shoombuatong, H.-C. Lee, J. Chaijaruwanich, H.-L. Huang, and S.-Y. Ho, "SCMCRYS: predicting protein crystallization using an ensemble scoring card method with estimating propensity scores of P-collocated amino acid pairs," *PloS one,* vol. 8, no. 9, p. e72368, 2013.

[2] H.-L. Huang *et al.*, "Prediction and analysis of protein solubility using a novel scoring card method with dipeptide composition," in *BMC bioinformatics*, 2012, vol. 13, no. S17, p. S3: Springer.
